# COVID-19 outbreak: Impact on global economy

**DOI:** 10.3389/fpubh.2022.1009393

**Published:** 2023-01-30

**Authors:** Saira Naseer, Sidra Khalid, Summaira Parveen, Kashif Abbass, Huaming Song, Monica Violeta Achim

**Affiliations:** ^1^School of Economics and Management, Nanjing University of Science and Technology, Nanjing, China; ^2^Department of Management Sciences, Kinnaird College for Women, Lahore, Pakistan; ^3^Department of Obstetrics and Gynecology, Nishtar Hospital, Multan, Pakistan; ^4^Riphah School of Business and Management, Riphah International University Lahore, Lahore, Pakistan; ^5^Department of Finance, Babes-Bolyai University, Cluj-Napoca, Romania

**Keywords:** COVID-19, pandemic, sectors, mitigation strategies, global economy

## Abstract

COVID-19 has been considered the most significant threat since World War II and the greatest global health disaster of the century. Wuhan City, Hubei Province, China, reported a new infection affecting residents in December 2019. The Coronavirus Disease 2019 (COVID-19) has been named by the World Health Organization (WHO). Across the globe, it is spreading rapidly, posing significant health, economic, and social challenges for everyone. The content of this paper is solely intended to provide a visual overview of COVID-19 global economic impact. The Coronavirus outbreak is causing a global economic collapse. Most countries have implemented full or partial lockdown measures to slow the spread of disease. The lockdown has slowed global economic activity substantially, many companies have reduced operations or closed down, and people are losing their jobs at an increasing rate. Service providers are also affected, in addition to manufacturers, agriculture, the food industry, a decline in education, the sports industry, and of entertainment sector also observed. The world trade situation is expected to deteriorate substantially this year.

## 1. Introduction

Wuhan, China, reported 27 cases of novel pneumonia on December 31, 2019. The cause and origin of the novel pneumonia were unknown. Wuhan city, Hubei province, is a trendy industrial hub in central China, having a population of more than 11 million (1). Novel pneumonia patients experienced fever, dyspnea, and lung infiltrates on imaging and dry cough. The spreading of new unknown pneumonia related to Wholesale Seafood Market Wuhan, famous for trading bats, crocodiles, dogs, pigs, fish, snakes, marmots, and wild animal species poultry animals (2). A swab of the throat, taken on January 7, 2020, was analyzed by the Chinese Center for Disease Control and Prevention (CCDC) and was identified as Severe Acute Respiratory Syndrome Coronavirus 2 (SARS-CoV-2). This disease is classified as COVID-19 (3). COVID-19 was designated a worldwide health emergency on January 30, 2020, with poor health systems most in danger.

The WHO didn't name the Chinese outbreak a pandemic, but it did call it a public health emergency. Amid the uncertain situation in Wuhan due to Cov-19, international trade and supply chains have been suspended, asset prices have been upended, and the decision-making process for multinational companies is challenging due to limited information. Central China's financial hub has been identified as Wuhan. Several of the world's 500 best companies are headquartered in Wuhan, including SAP, Microsoft, and Group PSA (4). In addition to being the country's most prominent steel and car producer, it is also a major trade and transportation hub. Economic development has outpaced China's national growth in recent years, with its GDP growing by 7.8% in 2019, rather than the national average of 6.1% (5). In response to the spread of this novel virus, business activities temporarily ceased, and numerous international firms evacuated their workers. The strict travel restrictions in Wuhan and different urban communities in Hubei are expected to harm global trade in China. Tourism, retail, and hospitality will all be negatively affected (6). More than 70 thousand cinema was closed in China (7); several airlines canceled flights from and to China, disrupting tourism and other activities. It is already beyond the Hubei borders that the novel virus is harming the economy. This influence severely causes stock markets to crash (8–10). The lockdown of Hubei province, with a population slightly smaller than the U.K. and France, threatened to hit the global economy was very exciting. Due to China's complete integration, after the United States, China is the second-largest economy, then the rest of the world. Global economic losses of $40 billion were estimated as a result of the SARS epidemic in China from 2002 to 2003 (11).

**Figure 1:**
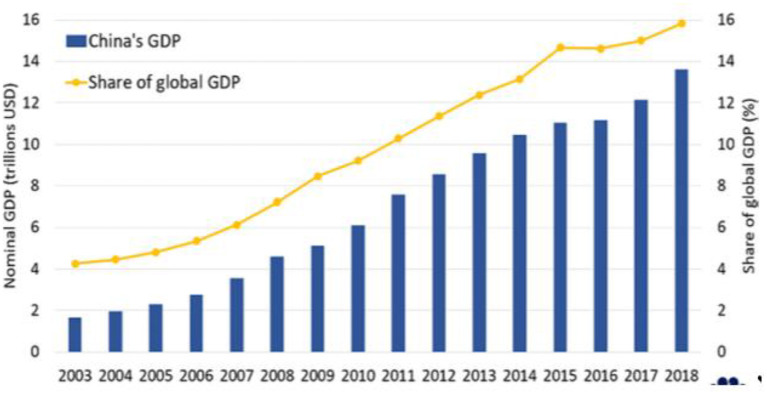
GDP growth in China and China's share of the global economy. Source: World Bank, OECD.

Currently, China's economic scale is 8–9 times higher than the epidemic of SARS. Financial experts worldwide say that the impact of COVID-19 on the world economy will be considerably vast in 2019. In 2019, China was estimated to contribute 39% to the global economy by the International Monetary Fund. Currently, China contributes 16.3% to the worldwide economy (12). It is suggested that the global economy was go-down if the nation's economy drops (13). As shown in Figure 1, the Chinese economy has grown considerably from 2003 to 2019, contributing to the global economy.

Due to the Lunar New Year holiday's extension, a sharp drop was seen in market value when China resumed its important businesses and industries on Monday, February 3, 2020. There was an 8.4% decline in the market value of the Shenzhen Composite, and a 7.7% decline was experienced by the benchmark Shanghai Composite, which is approximately a $375 billion decline in market value since August 2015; it is the sharpest 1-day decline in market value. The decline was also experienced in other businesses, such as transportation stocks, consumer services, and retail companies (14).

The 11 provinces of China, under strict control then, produced two-thirds of all the auto, the country's vehicles. Chinese auto parts are manufactured in these provinces, especially Hubei, for the South Korean market, Europe, and the United States. The experts believe there could be about 350 000 unit's production loss that might be possible if the production companies and manufacturing industries remain inactive until February 10, 2020. If the outbreak continues till the mid of March, this loss may be reached over 1.7 million, and it is expected that this decline could be reached up to 32.3% (15). Similarly, Standard & Poor's (S & P) believes that auto parts production in the United States may decline by 50%. Some automakers have expressed concerns about the shortage of auto parts. A lack of features in China has forced the Hyundai Motor Company to close its domestic factories. Automakers in the United States and Europe are similarly concerned that a shortage of spare parts could disrupt their business (16). Several industry analysts have forecast that China's automotive market will shrink. If the coronavirus outbreak continues into the second quarter, it will reach 3–5% in 2020.

According to the United Nations Tourism Organization (UNWTO) (17), China was a significant tourist source market during this outbreak and a major tourist destination. In 2019, approximately 6.3 million Chinese tourists traveled abroad for Lunar New Year, generating about US$73 billion in revenue. However, this number declined significantly in 2020 (18). According to reliable sources, this also substantially impacts tourism outside of China. Vietnamese tourism is expected to lose up to $ 7.7 billion in the first quarter due to cancellations of Chinese groups and the general economic downturn. In 2020, there will be an 80% decrease in Chinese tourists visiting Thailand, reducing Thailand's revenue by the U.S. $ 3.1 billion (19). Indonesia, Singapore, South Korea, Malaysia, Cambodia, Hong Kong, Japan, Australia, and other nations will be affected, in addition to Vietnam and Thailand (20).

**Figure 2:**
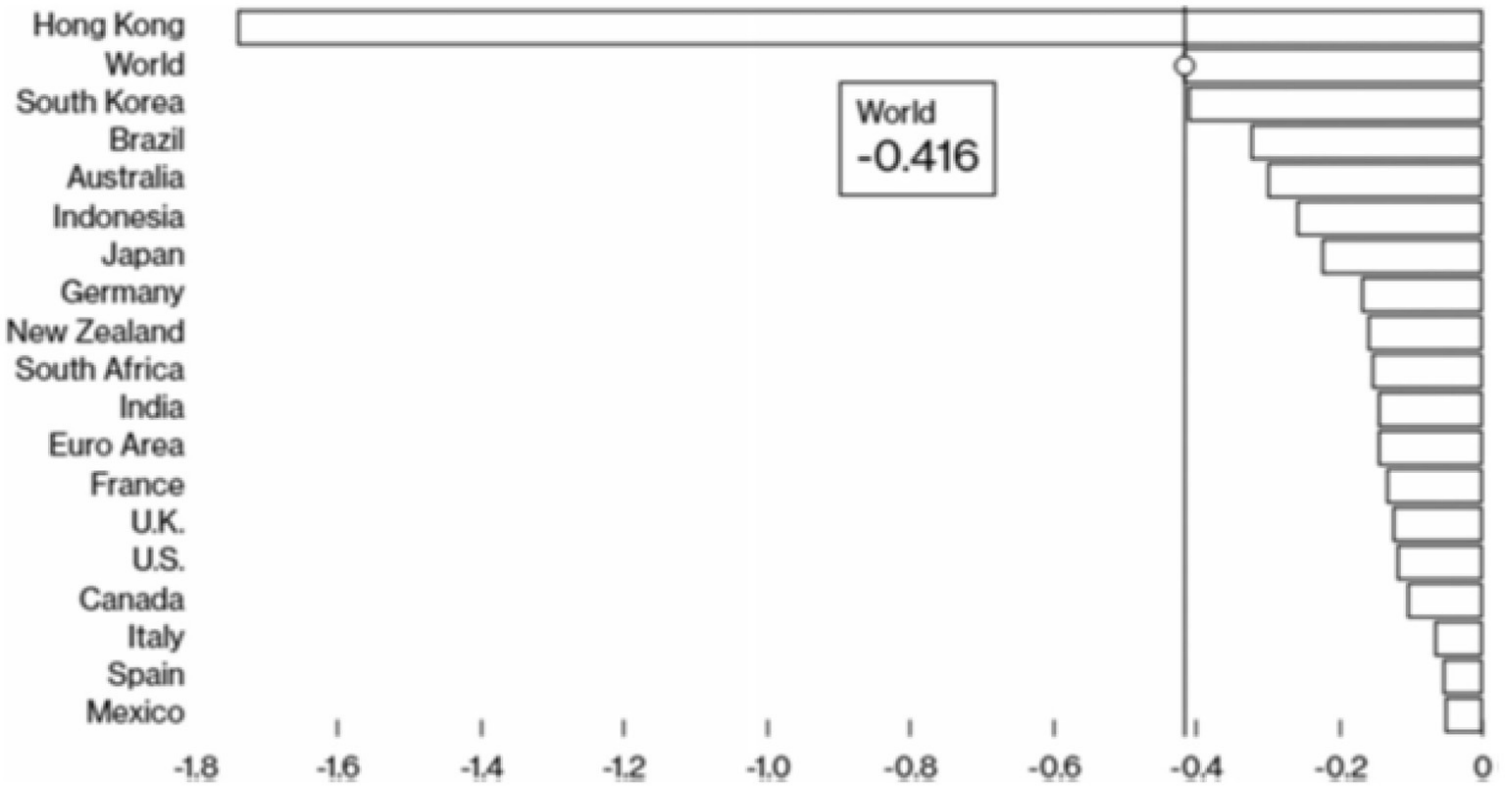
Growth deviation in percentage points from Q1 2020. Source: Bloomberg economics.

The economic consequences of this outbreak are enormous in China and even around the world, considering the loss of trade, and tourism, rise in unemployment, industry recession, a decline in sustainability and quality of life, the decline in the education sector, and the effect on the agriculture industry, impact on the food industry, the fall of the sports industry, deterioration of the entertainment sector, and aspects of the global supply chain. In the first quarter, Bloomberg Market Diagnosis predicts China's GDP growth will fall by 4.5%. Global GDP is expected to decline by about 0.42% in the first quarter due to the outbreak, as shown in Figure 2 (21).

## 2. Methodology

**Figure 3:**
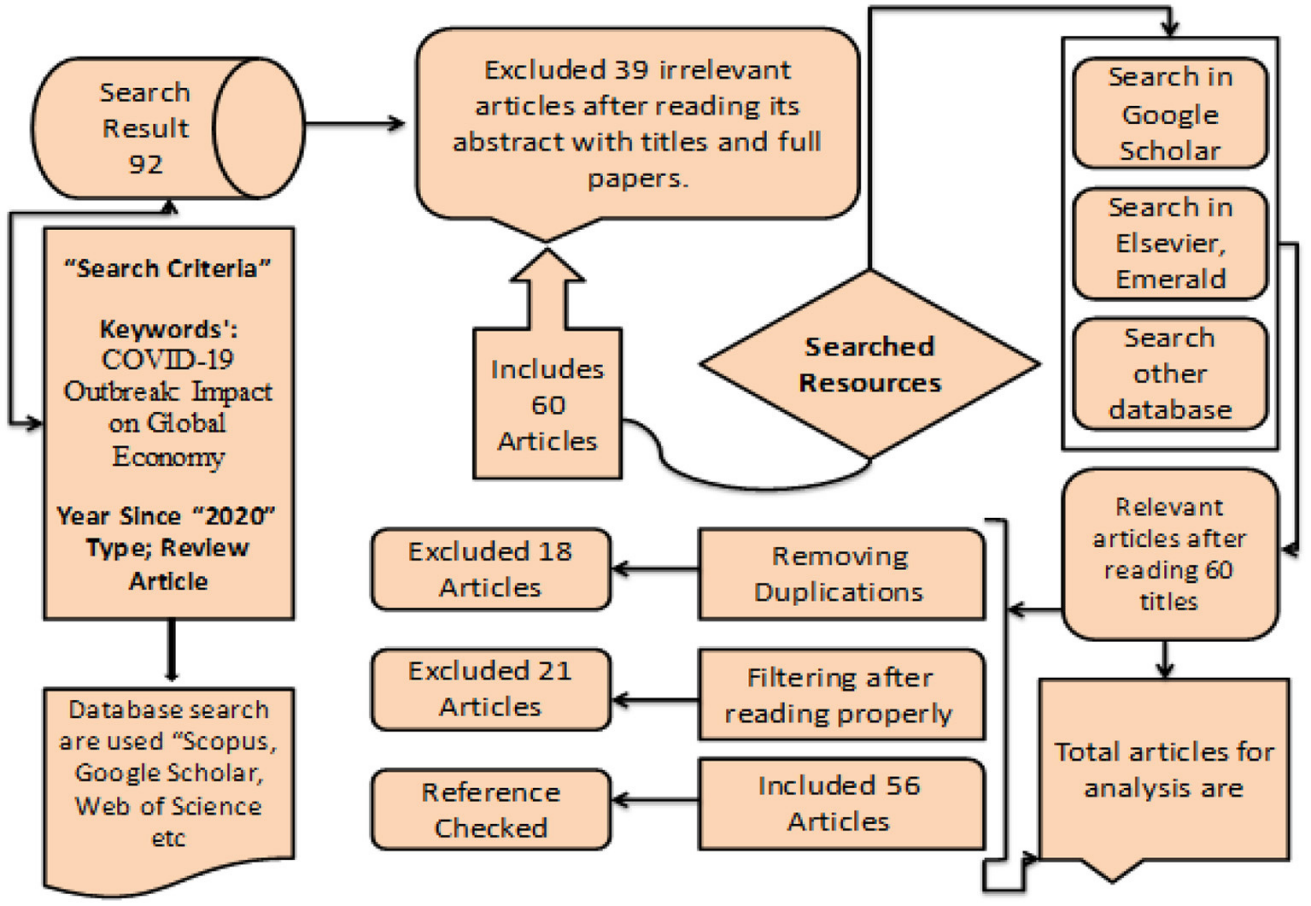
Methodology search for finalized articles for investigations. Source: Author's constructed.

The research mainly included reviewing previous studies and determining COVID-19's global economy hit. According to experts, the virus disrupted global supply chains and closed industries. This interruption impacts worldwide demand and production. The research methods included a review of previous studies, determining global recession due to the natural disaster, and determining vital economic indicators, such as the rise in unemployment, the industry hit hard by the recession, the slump in manufacturing activity, a bad year for trade, the global economy shrinkage in 2020, the decline in Sustainability and Quality of Life, the decline in Education Sector, resultant Effects on the Agriculture Industry, and reduction in the entertainment sector. In addition, as explained in Figure 3, the search approach was used for this research topic. In this study, many research databases were consulted to search for relevant papers and download them from the database (Google Scholar, Scopus Index Journals, Emerald, Elsevier Science Direct, Springer, and Web of Science). Our primary emphasis was on items published in academic publications, including research articles, feedback pieces, brief remarks, discussions, BBC news, world bank source, and review articles. The reports were utilized to search for numerous keywords, such as “impact of COVID-19 internationally,” “impact of COVID-19 on a macro level,” and “spillover effects of COVID-19 on micro-level globally,” etc.; in summary, a keyword list and the complete text have been created. In the beginning, searching for keywords produced a significant volume of published material.

Since 2020, it has been challenging to assess all papers; the literary display has constraints. The research searched 92 articles in the database mentioned above. It removed 39 irrelevant publications since they were copied from a previous search. The framework consists of two things:

(i) Articles focused on “Global COVID-19 Impacts, impact on the global economy, and sustainable mitigation strategies.”
(ii) Search phrases linked to study requirements.

**Figure 4:**
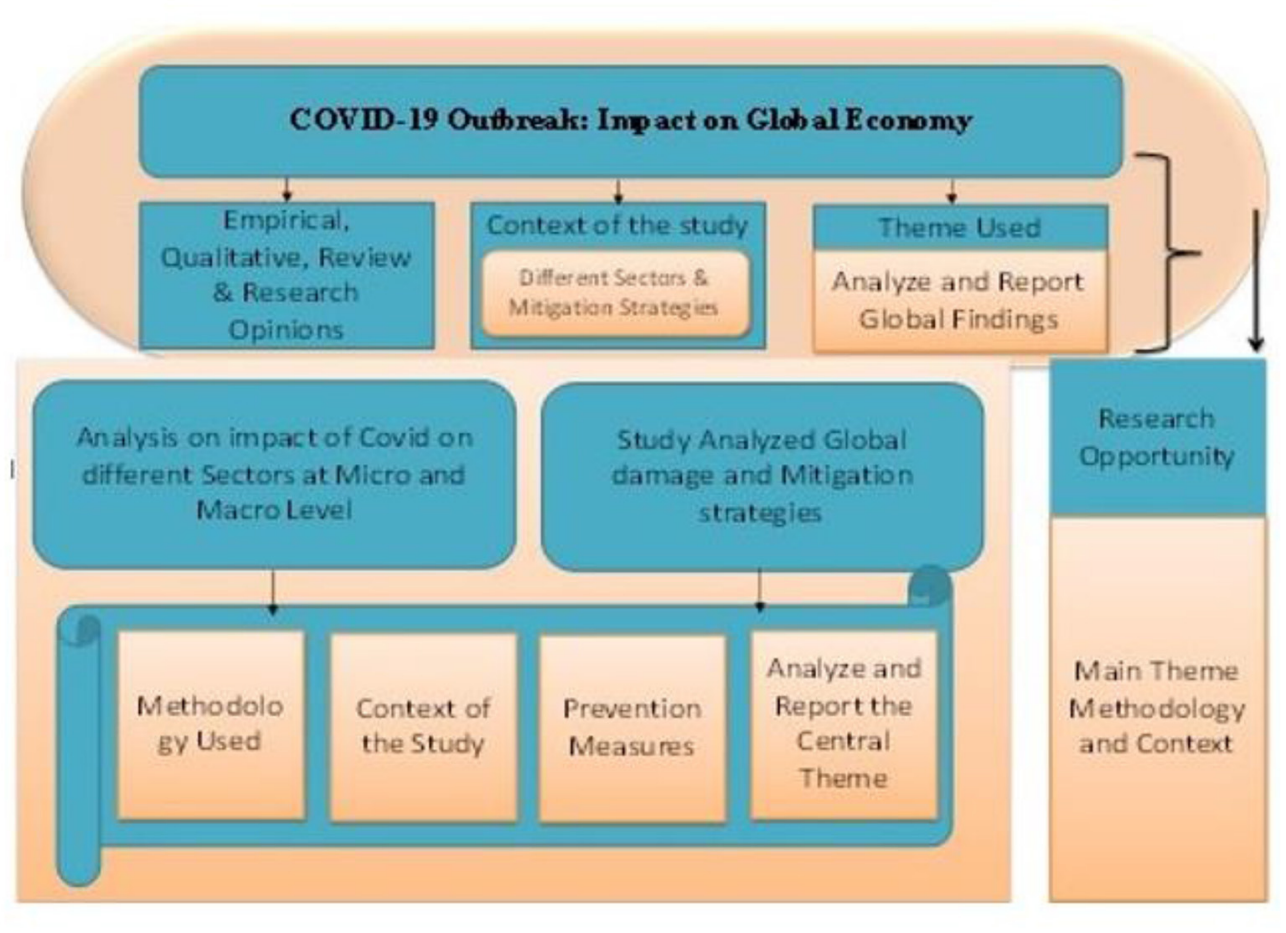
Framework of the study. Source: Author's constructed.

Our search produced 60 articles. We scan “Web of Science and Google Scholars” to improve search results and verify cited papers. This study analyses 60 papers' research subjects, techniques, settings, and theories explained in Figure 4. This study explores connected domains to provide future research prospects.

## 3. The global economy hit by coronavirus pandemic

Johns Hopkins University data shows that as a result of the coronavirus, 270 million people have been infected, and 190,000 have died. Several countries and cities worldwide have prevented the virus from spreading further. Some measures include closing borders, shutting down schools and workplaces, and prohibiting huge gatherings. Global economic activity was halted by the “Great Lockdown” due to the global financial crisis, harming businesses and causing job losses. “We are facing a real global crisis since no country is immune,” IMF chief economist Gita Gopinath wrote earlier this month (22). Some facts and graphs illustrate how the Coronavirus epidemic has affected the global economy.

### 3.1. The rise in unemployment

**Figure 5:**
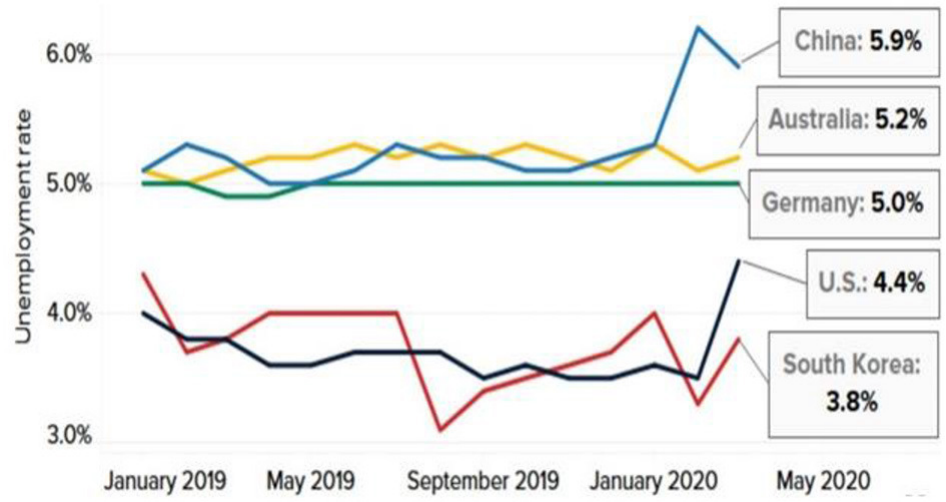
Coronavirus padamic hit jobs. Source: Bureau of Labor Statistics of the U.S.; National Bureau of Statistics of China, Deutsche Bundesbank, Australian Bureau of Statistics, Statistics Korea, Refinitiv.

In some economies, the number of unemployed has increased due to global lockdown measures, as shown in Figure 5.

In the U.S., the world's biggest economy, almost 26 million jobs have been lost in the previous 5 weeks (23). Unemployment in March topped 4.4%, the highest level since August 2017. It is not limited to the United States. There has also been increased unemployment in Australia and South Korea, and some economists believe this trend will continue.

### 3.2. The industry hit hard by the recession

**Figure 6:**
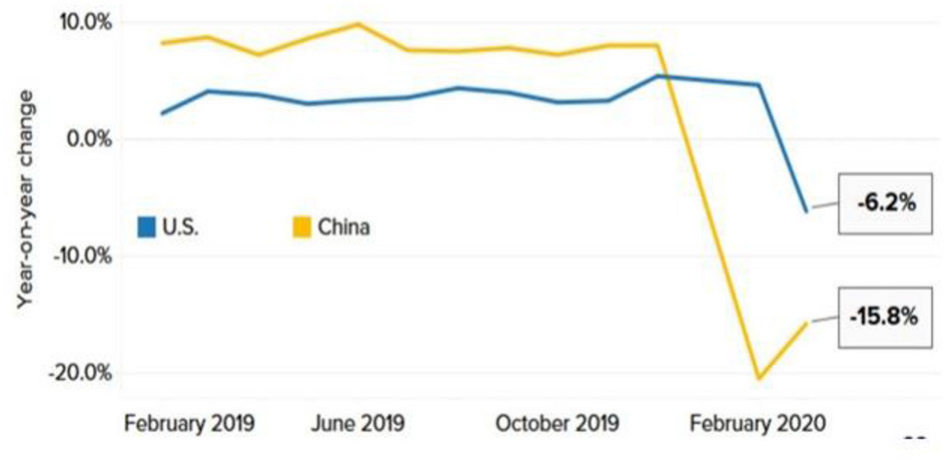
Plunge in retail sales. Source: U.S. Census Bureau, National Bureau of Statistics of China, Refinitiv.

Numerous significant economies, notably the United States and China, depending on the service industry for employment and economic development. During the Pandemic, many stores closed, and customers stayed home due to a lockdown imposed by both countries. Amazon and some other retailers reported an increase in online sales, but this did not stop the decline (24). Despite lifting the locking measures, economists warn that consumers may not regain their consumption. Even after China allowed companies to reopen gradually, analysts at the Oxford Economic Research Institute observed that China's retail industry did not improve rapidly, as illustrated in Figure 6.

**Figure 7:**
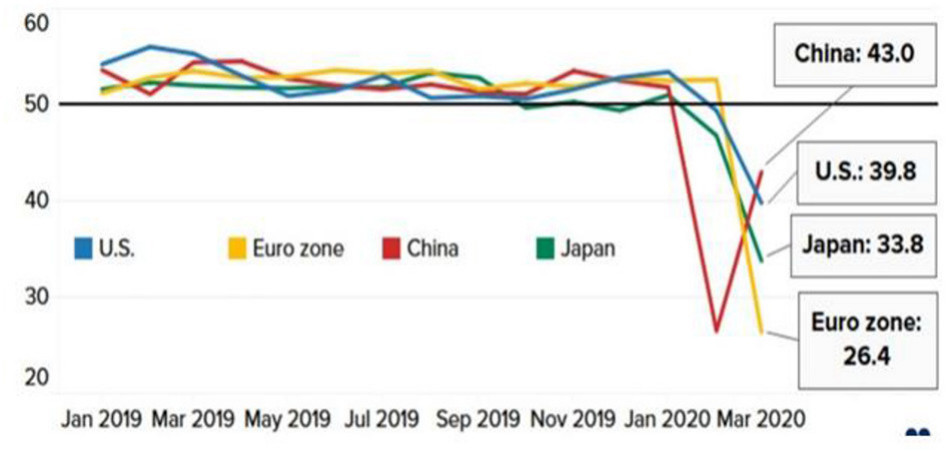
Services activity in major economies. Source: IHS Market, Caixin, au Jibun Bank, Refinitiv.

Reports indicate that consumer spending will not return immediately after restrictions are lifted due to the slow improvement in household spending. According to IHS Market, the global service industry, transportation, real estate, and business activities in the travel and tourism industries show significant decline (Figure 7).

**Figure 8:**
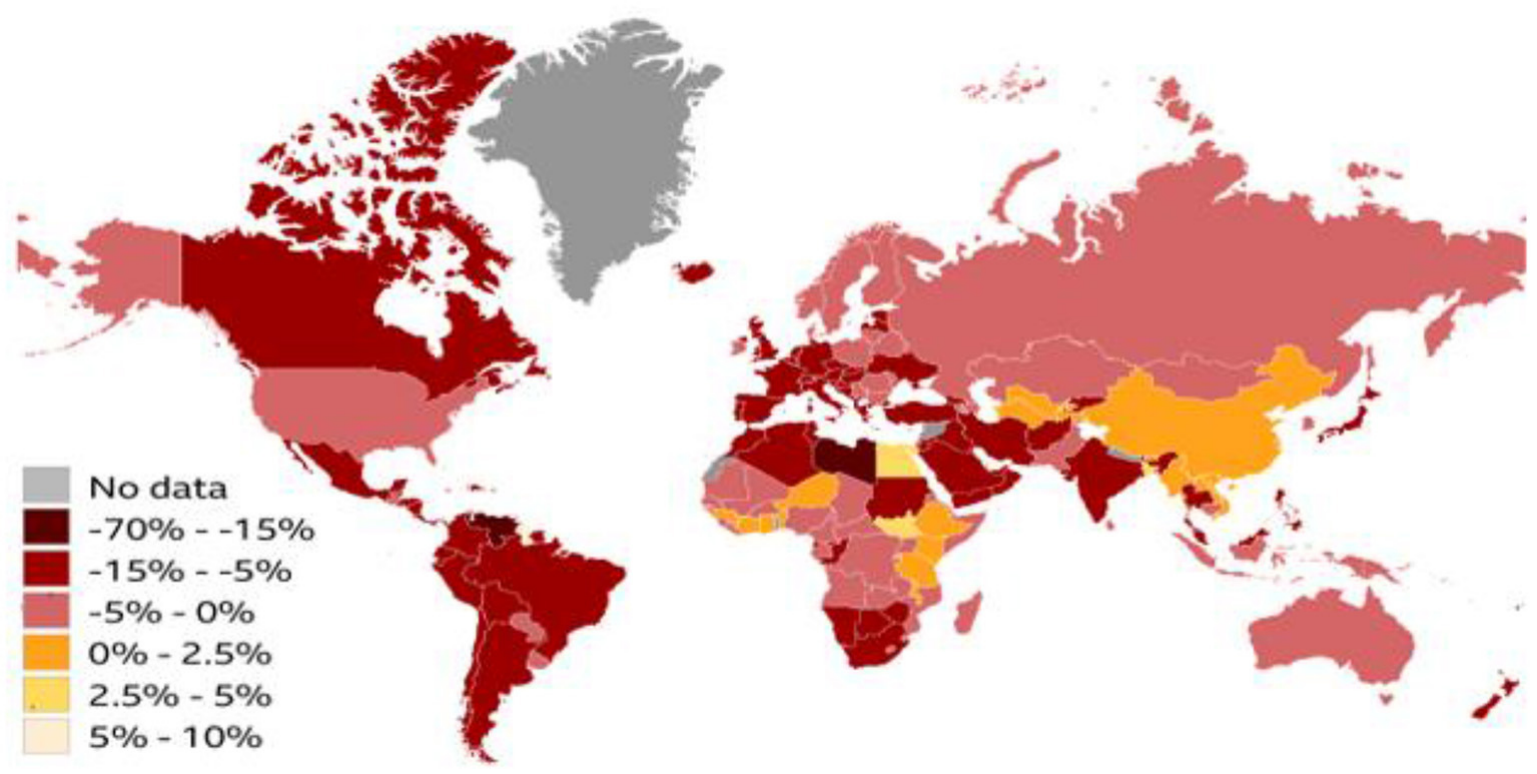
Majority of countries in recession. Source: IMF.

Growing economies tend to create more wealth and jobs. It is measured as the change in the overall value of goods and services produced over 3 or 12 months. In 2020, the IMF predicted a 4.4% contraction in the global economy. According to analysts, this will be worse than the Great Depression. Among the major economies, China was the only one to grow in 2020. Its growth rate was 2.3%. According to the IMF, global growth is expected to be 5.2% in 2021. India and China are expected to lead the way with growth of 8.8 and 8.2%, respectively. The recovery is expected to be slow in the U.K. and Italy, two big, services-reliant economies that have been hit hard by the outbreak (Figure 8).

### 3.3. The slump in manufacturing activity

**Figure 9:**
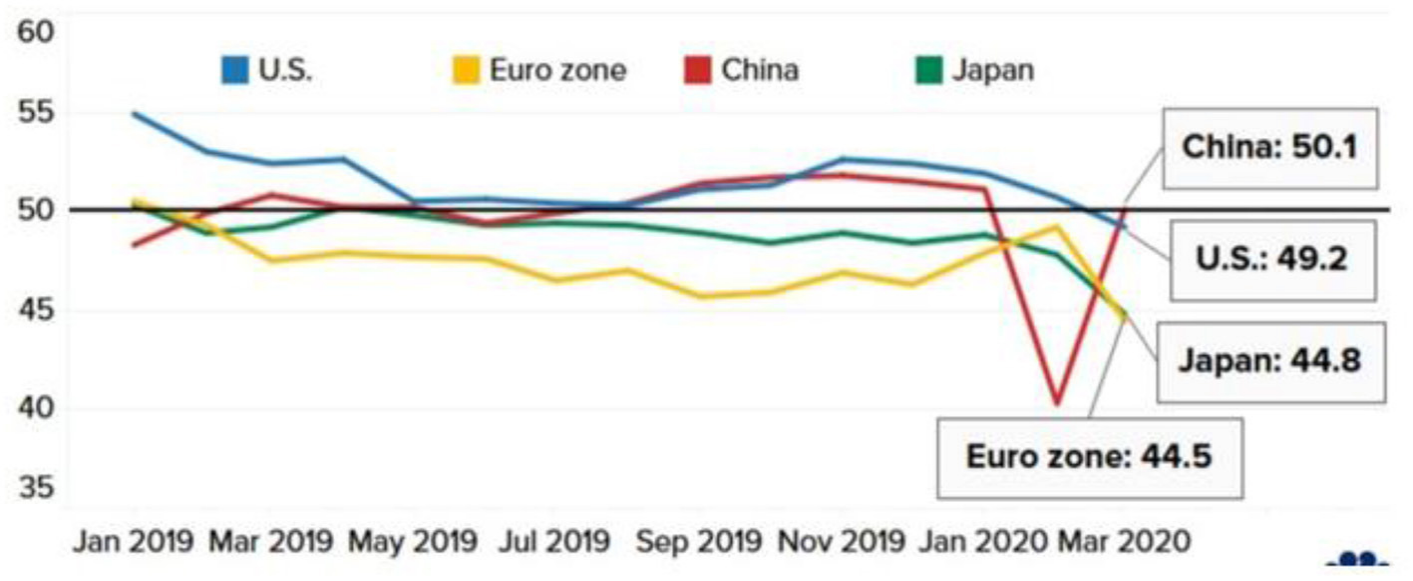
Manufacturing in significant economies. Source: IHS Market, Caixin, au Jibun Bank, Refinitiv.

Manufacturers have been impacted by the trade war between the U.S. and China for the past two years and have been under pressure again while the coronavirus spreads worldwide (see Figure 9).

**Figure 10:**
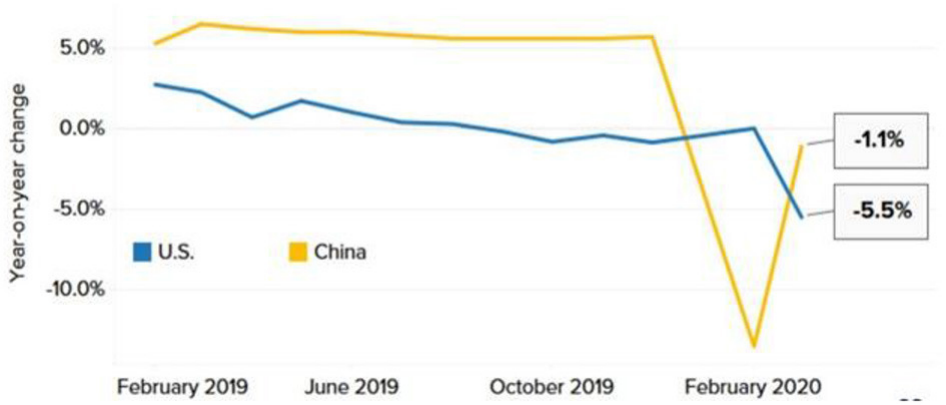
Coronavirus impact on factory output. Source: U.S. Federal Reserve, National Bureau of Statistics of China, Refinitiv.

Due to the COVID-19 epidemic, non-Chinese firms that received their raw materials or components from Asian vendors (sometimes known as “intermediate products”) were impacted. However, due to the authorities' efforts to contain the virus, the operation of the Chinese plant was suspended for longer than expected. Lockdown measures have hit more and more manufacturing companies as more and more countries implement them. A few are forced to close temporarily, while others are restricted from accessing intermediate materials and goods. In addition, a reduction in commodity demand has made manufacturing more challenging. As shown in Figure 10, the production of U.S. factories in Europe and Asia declined in the past month.

### 3.4. A lousy year for trade

**Figure 11:**
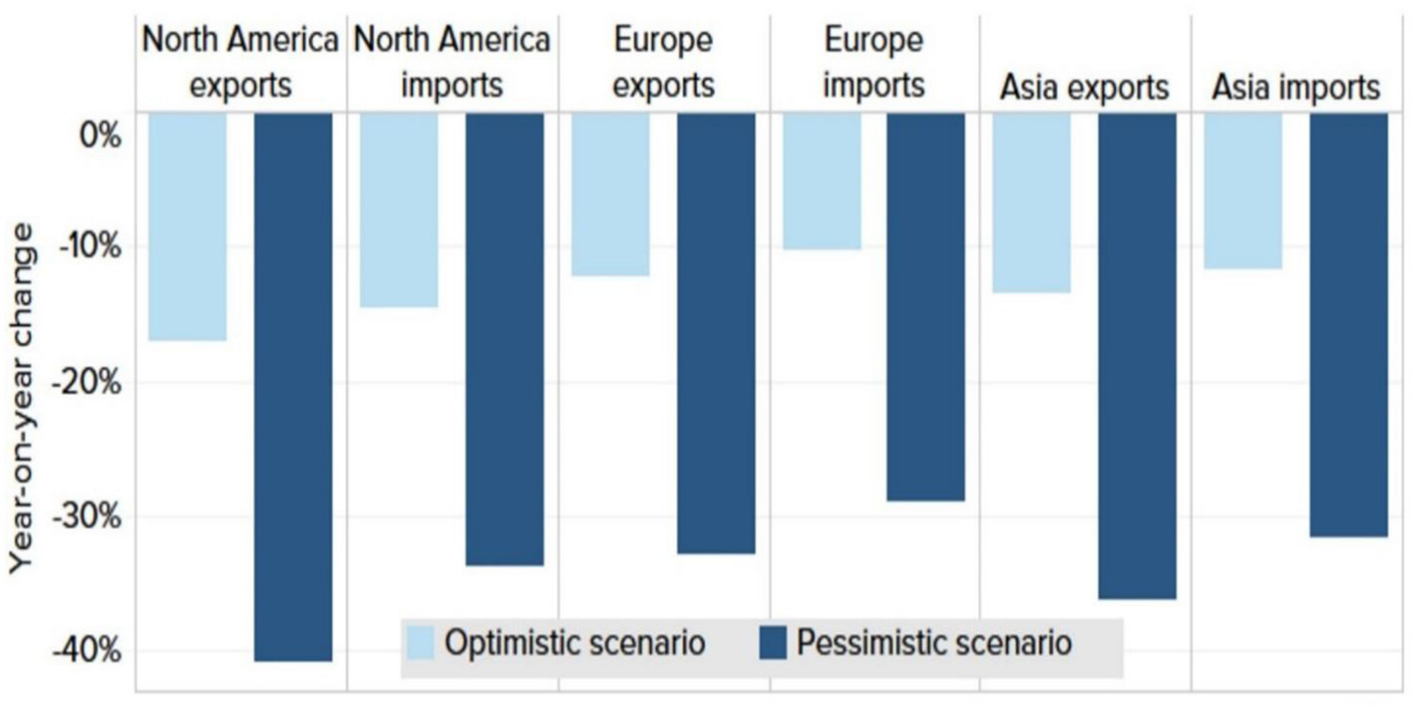
Decline in merchandise trade. Source: World Trade Organization forecast.

In 2020, it was expected that global trade would slow even more than it has in 2019. An anticipated decline in merchandise trade is shown below (see Figure 11).

The World Trade Organization forecasts that global trade could decline by 12.9 or 31.9% this year, depending on how global economic conditions develop (25). According to the WTO, imports and exports in all regions will decline by double-digits in 2020.

### 3.5. The global economy shrinkage in 2020

**Figure 12:**
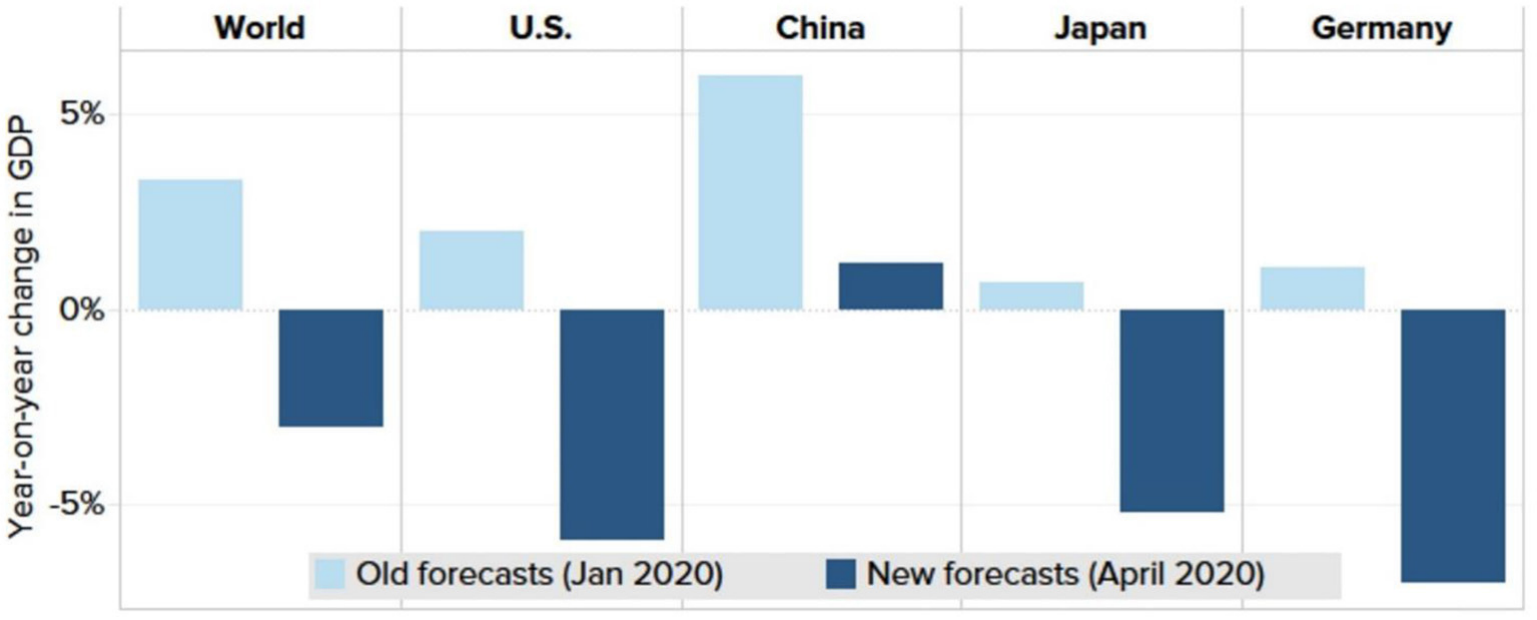
Economic forecasts (2020). Source: IMF World Economic Outlook.

Numerous institutions have drastically reduced their global economic forecasts due to the Coronavirus pandemic. Global economic contraction is expected to reach 3% this year, according to the International Monetary Fund (26). Figure 12 shows only a few economies (including China and India) will grow in 2020.

Although the economy is expected to bounce back next year, the recovery will only be a part of the reason because the level of activity will be lower than in 2021 before the virus hit” (27). In 2020 and 2021, the pandemic crisis may cause the global economy to lose nearly $9 trillion, more than Japan and Germany combined.” wrote the organization's chief economist Gopinath.

### 3.6. The decline in sustainability and quality of life

Politics, ecology, and the economy influence sustainable development (28). These factors also dictate how people should live so future generations may experience the same quality of life. According to Garfin et al. (29), tiny changes in human existence steadily impact the future. The future generations will not recognize the changes in their lives as a response to change; they will assume that individuals before them had comparable lives. The COVID-19 Pandemic has altered human existence's political, environmental, and economic elements, which affect psychological growth and sustainability. This impacts people's living standards and quality of life. The COVID-19 era resulted in social problems and international crises in the early 2020's (30). According to Arden and Chilcot (31), progress and development have halted, and the epidemic has disrupted the financial stability of industrialized and developing nations. COVID-19 measures such as social isolation and locking up reduce the quality of life because of the resulting stress and sadness (32).

Most people are losing employment and money worldwide (33). Profit margins and sales have fallen. Many nations have created methods to rescue their economies and populations' mental health from the epidemic, although regaining economic stability will take years. COVID-19 and related crises have harmed mental health, particularly among job losers. Employees' mental health is damaged by their unpleasant, contentious relationship with the company. The international economy has stopped, and millions have perished as COVID-19 caused unemployment, illness, and despair. The coronavirus triggered a large-scale psychological experiment that would impact life. Knowing that the Pandemic harms mental health reduces life quality and motivates prevention. Sustainable management improves worker wellbeing, according to Topa et al. (34). Anxiety, stress, and exhaustion affect behavior (35). Lockdown endangers lives; on the other hand, PTSD, hunger, and mental issues are rising. COVID-19 kills people.

### 3.7. The decline in education sector

Coronavirus outbreak affects global schooling. Due to the coronavirus outbreak, several educational institutions closed worldwide (“Impact of Coronavirus Pandemic on Education,” 2020). According to Education UNESCO (36), over half of the world's students were affected by Worldwide closures observed by the United Nations Educational, Scientific, and Cultural Organization (UNESCO). This event severely disrupted many students' academic and professional ambitions; consequently, coronavirus damaged over $600 billion in businesses. In 2020, educators and students throughout the globe felt the rippling impact of the coronavirus as institutions and universities were urged to close.

On the whole, 44 countries on four continents had school cancellations, affecting hundreds of millions of pupils. Schools without an online learning platform were disproportionately affected. Moody's lowered the U.S. higher education outlook from stable to “negative” because 30% of U.S. colleges and universities already had inadequate operational performance, and it was difficult for them to react to the financial and academic reforms needed to deal with the coronavirus pandemic (37). As the coronavirus pandemic developed seriously in Italy, France, and Spain, several U.S. universities urged study-abroad students to return home. However, the epidemic encouraged online and remote learning, while only a minuscule proportion of the world's education is taught online (38). COVID's worldwide influence was considerable, according to UNESCO data showed that by 2021. The COVID pandemic impacted around a 10.5 million learners worldwide, or 0.7% of all registered students, yet there was only one country-wide learning shutdown by 2021 (39). In the first quarter of 2022, 6 country-wide closures and 43.5 million impacted students, or 2.8% of all registered students. This caused students to have more debt, take longer to graduate, and break their academic dreams during COVID-19 from 2020 to 2022 (40).

### 3.8. Resultant effects on the agriculture industry

Due to the COVID-19 epidemic, the resiliency of the agriculture industry has been put to the test. The cost of agricultural goods has decreased by 20% due to a worldwide slump in demand from hotels and restaurants. Nations have implemented several preventative measures over the globe to halt the rapidly escalating spread of the disease (41). This involves separating oneself from their social circle, minimizing the amount of needless travel one does, and not attending any churches. If people are urged to self-isolate after contacting suspected virus carriers, the number of available inspectors and delivery workers may be affected (42). This will have significant repercussions for perishable products, such as food that is high in fat and protein, such as meat and vegetables. In addition, markets have taken matters into their own hands by prohibiting floor trading, which has harmed the capability of commodities exchange (43). One example from more recent times is the Chicago Mercantile Exchange. “Panic purchasing” complicates shortages beyond what's available in supermarkets. Concern has been voiced by the American Veterinary Medical Association (AVMA) over the inadequate supply of animal medications made available by several prominent drug suppliers.

### 3.9. The decline in travel and tourism

The hotel business has also suffered from COVID-19's travel ban. Community lockdowns, social isolation, stay-at-home orders, and travel and mobility limitations have led to the temporary closure of numerous hospitality enterprises and diminished demand for those permitted to continue operating (44). The coronavirus has affected hospitality the most. Hourly employees in the hospitality and tourism industries faced severe difficulties due to the economic downturn. Marriott International, which has around 174,000 employees, furloughed tens of thousands of those people. In addition, Hilton Worldwide informed its lenders on March 5, 2020, that it would be borrowing a precautionary $1.75 billion as part of a revolving loan (45). This move was made to save money and to keep the company's flexibility” in light of the volatility in the global markets. The U.S. hotel industry's income per available room declined by 11.6% on March 7, 2020, while China's occupancy rates plunged 89% by January 2020 (44). Other U.S. hotel corporations want $150 billion in direct employee help, owing to a drop in demand and a $1.5 billion loss since mid-February (46). Since March 1, 2020, German hotel occupancy has dropped 36%. Rome's occupancy rate is 6%, whereas London's is 47% (47). The COVID-19 crisis has caused worldwide distortions in the hospitality sector and the European hotel market slumps.

### 3.10. Effects on the food industry

Due to consumer purchasing during COVID-19, the food industry has been under much pressure, particularly in food delivery and retailing. The food supply chain affected farmers, distributors, consumers, and labor-intensive food-processing businesses. Many factories restricted, halted, or temporarily ceased production owing to COVID-19 employees who were unwilling to go to work, assuming they would become sick at work, mainly in meat-processing food firms during the epidemic (48). Because of this, there was a growing fear that there may be a scarcity of food, and panic purchasing has led to an increase of £1 billion worth of food stored in households throughout the United Kingdom (49). The rising demand for food goods has also impacted online meal delivery. In addition, food banks have been affected by people shopping in a panic and storing food in response to the decline in contributions (50). Despite government assurances, businesses have made substantial adjustments, including limiting the number of products consumers might purchase, creating more than 30,000 new jobs to refill shelves, and introducing special shopping hours for the elderly, vulnerable populations, and NHS goods (51).

### 3.11. The decline of the sports industry

COVID-19 influenced the scheduling of athletic events, including some of the biggest competitions in the sport's history. The highly anticipated football event, Euro, was pushed back a year, and the playoffs won't occur until at least June of the following year (52). The athletes and their nations agreed to postpone the games until 2021. Many tournaments, including the British Open, were canceled or moved. Pandemic cancellations cost billions (53). Tokyo's Olympics and Paralympics were postponed. The 2020 English hockey games were postponed. MLR's 2020 season was canceled, and all MLB season games in Mexico and Puerto Rico were canceled (54).

Due to the Portuguese government's declaration of an emergency, all sports activities will be postponed until further notice. The World Snooker Championship scheduled to take place in Sheffield has been delayed (55). The 2020 European Aquatics Championship in Hungary was postponed until August. Sponsors and organizers of the aborted games lost billions. In 2021 and 2022, numerous nations restarted sports, but the number of participants at sports arenas was limited, and face masks were required.

### 3.12. The decline of the entertainment sector

Pandemic affected cinema, theater, and entertainment. The 2020 coronavirus pandemic cost the entertainment industry $5 billion in losses, and several Hollywood films delayed their release. Consequently, most of the 120,000 below-the-line entertainment jobs lost were stage roles. The shutdown lost 150,000 members and 120,000 jobs, and the IATSE requested the government to include the entertainment industry in its stimulus package (56). From February 23 to March 1, 2020, the COVID-19 epidemic in Italy cost millions of euros every week. Film screenings, theaters, live music, dancing, and exhibits lose money (Khan et al., 2022). Collectively, unemployment in the entertainment business surged to new highs, but it was uncertain whether it would receive government stimulus funding. Some senators contended that the entertainment business contributes less to the economy than the finance and industrial sectors (57). Entertainment activities resumed in 2021 and 2022, but the industry's performance didn't approach pre-COVID levels due to weak economic recovery. 221,762,724 and 498,162,785 tickets were sold in 2020 and 2021, compared to 1,314,169,169 and 1,225,910,803 in 2018 and 2019. COVID reduced the number of moviegoers by 83%. In 2020 and 2021, box office revenue was $2,033,566,047 and 4,568,154,099. In 2018 and 2019, it was $11,972,083,658 and 11,229,345,558. Film revenue plummeted by 83.1% under COVID over pre-COVID (58).

## 4. Challenges and mitigation strategies

The suggested study approach advises concentrating on economic policies that have extended the medical crisis and combining monetary, industrial, and other sectors to settle worldwide and financial market issues. Figure 11 illustrates the study's structure and topics.

### 4.1. Policy challenges

Economic analysts have used aggressive strategies to handle short-term irregularities in nations that can endure the outbreak without fabrications. The rapid evolution of the global health catastrophe into international corporate trade and economic calamity has left experts throughout the globe stunned. As the epidemic's fiscal effect develops, analysts concentrate more on immediate monetary repercussions than longer-term factors like debt accretion. Due to the poor elasticity of budgetary and financial assistance under conservative criteria, many strategists have limited their ability to react to the present crisis, given the recent drop in global economic growth, mainly production and trade, since the flu pandemic began influencing international policymaking. Initially, the Pandemic's fiscal effect was anticipated to be a temporary supply of items as employees self-quarantined *via* social interaction to reduce the outbreak's breadth. As corporations store cash, these growing financial impacts may create liquidity and credit limitations in international monetary markets and hurt economic development. Some viewers wonder whether these economic undercurrents portend full-scale worldwide financial difficulties.

**Figure 13:**
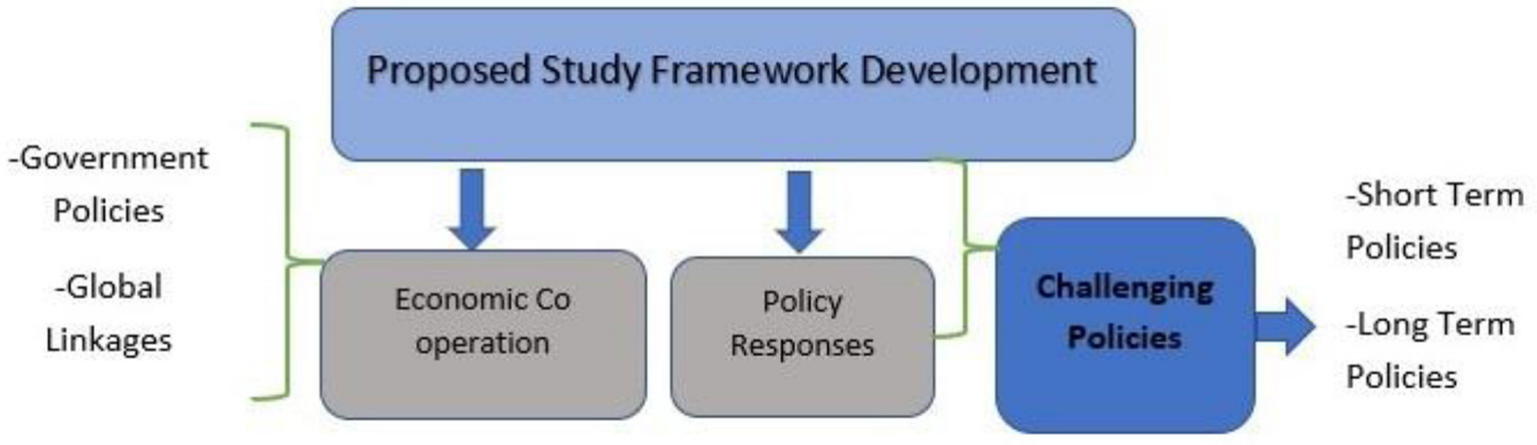
The proposed study framework for significant development. Source: Author's development.

According to the World Bank's Global Economic Prospects report from June 2021, some impoverished countries and rising economies are still battling COVID-19. Two-thirds of emerging markets and developing economies won't recoup per capita income losses by 2022. The Pandemic has reversed poverty-reduction benefits and created insecurity in low-income nations where vaccinations have lagged. Many countries had already pledged or implemented steps to help epidemic-affected economies. These nations decided on the kind of help to give (loans vs. direct payments), the amount needed, and how to define resources and financing conditions if any. Several governments have taken surprise fiscal and monetary measures to fight the crisis (59). These economies lack financial resources, and the medical care system is overworked and vulnerable. The downturn or upcoming recession harms all nations; thus, global leaders must overcome COVID-19's consequences on their economies quickly and aggressively. Therefore, according to Fernandes (60), global action is required to lay down a proposed framework with international cooperative macroeconomic policies to restore confidence and address the feasible solution to the post-COVID crisis barriers to overcome the expected great recession by using collective action with the following research questions (Figure 13).

- How can governments negotiate and cooperate to overcome severe revenue declines, strains on public budgets, and the prospect of sovereign evasions and a 2021–2025 recession?

**Figure 14:**
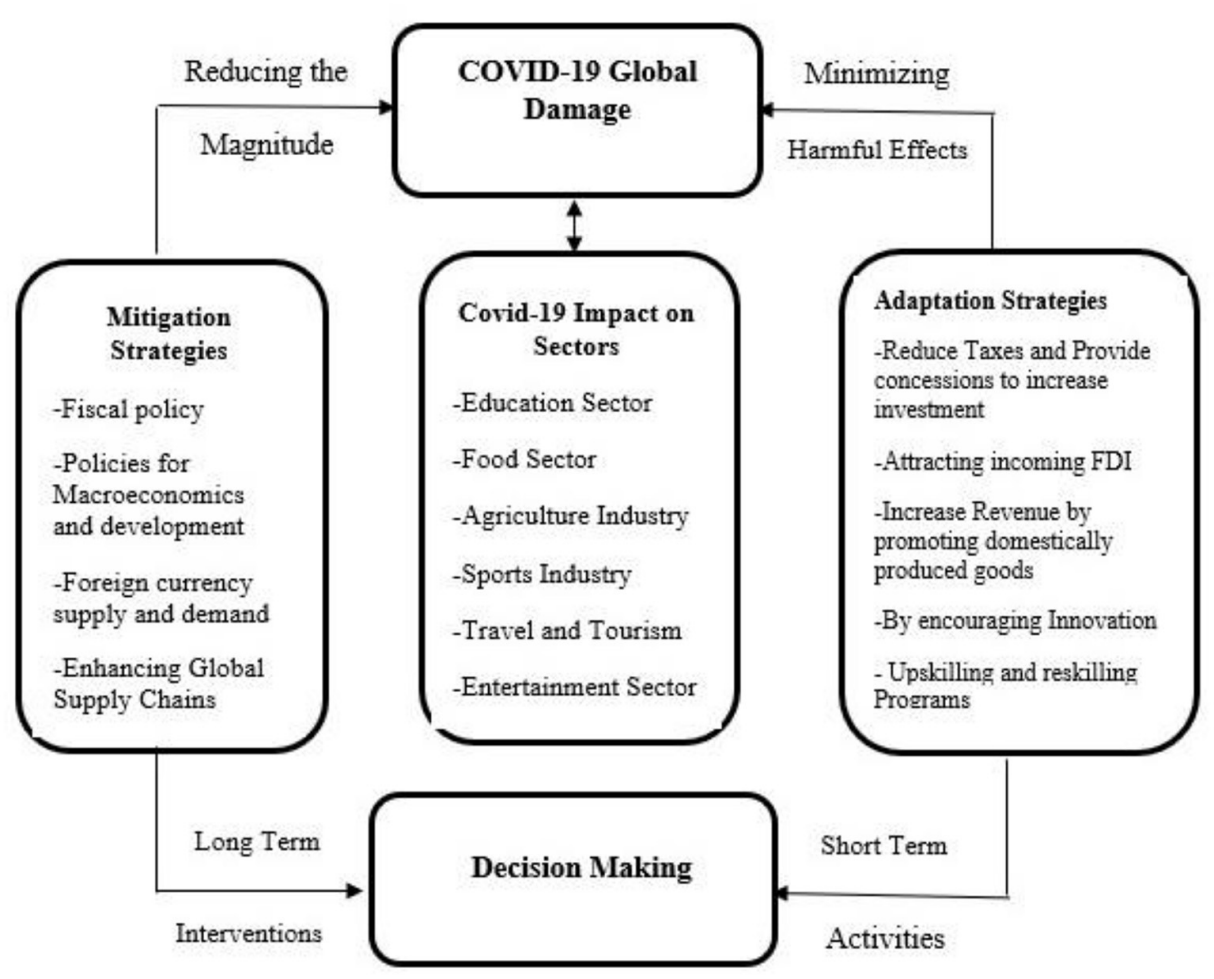
Sectorial impacts of climate change with adaptation and mitigation measures. Source: Author's constructed.

### 4.2. Mitigation strategies

Immediate and decisive policy actions are required not just to control the epidemic and save lives but also to safeguard the most vulnerable members of our society from economic disaster and maintain economic development and financial stability factors shown below in Figure 14.

### 4.3. Fiscal issues

Wealthy nations have launched massive health and public expenditure measures to combat the COVID-19 epidemic. Poorer countries have fewer fiscal and monetary alternatives, and international organizations may aid. Most wealthy countries have ramped up expenditures and used monetary policy to soften the shock of lockdowns and other measures that have closed enterprises and left many jobless. But underdeveloped nations, beginning to react more vigorously, may have fewer alternatives. Plan, coordinate, and execute a business continuity plan for COVID-19. According to Building a Resilient Recovery: How We Can Emerge Stronger from the COVID-19 Pandemic, Ensure revenue collection and agency operations to fund and manage crisis responses.

### 4.4. Policies for macroeconomics and development

National authorities and multilateral organizations worldwide are exploring extraordinary policy steps in response to the rising health crisis and dismal economic prospects. Central banks in developed and developing nations slashed interest rates, injected liquidity, and provided emergency financing for enterprises and families. About 60 monetary authorities have scored rates since the crisis began, typically in emergency meetings (61). Direct wage or income assistance measures may mitigate short-term socioeconomic consequences while retaining recovery capability. Tax deferrals, government-subsidized short-term labor, mortgage moratoriums, and immediate cash handouts are examples. Social assistance programs must target the elderly and those in vulnerable jobs during the crisis.

### 4.5. Manage vital supply and demand

Addressing fundamental supply and demand concerns may avoid shortages, price surges, and misery. Food and medication manufacturing and delivery need reliable transportation, energy, and communications. The crisis-management agency must form committees with the private sector and critical operators to assess daily the flow of essential products and services and the health of employees and vital people (62).

### 4.6. Foreign currency supply and demand

There is a good chance that the governments will have to limit foreign currency transactions to stop a run on the local currency caused by a rise in the amount of money in circulation. This is because a rise in the amount of money in circulation directly causes an increase in circulation. For the government to manage its foreign reserves effectively, it must be able to compute the cash flow required to fund the importation of food, medicines, energy, and other essential commodities for at least 6 months while also considering the flows of external debt.

### 4.7. International organizations have a significant influence

International organizations have authority in mediation, conflict settlement, peacekeeping, and penalties. They assist global health and monetary policies (63). COVID-19 showed how important international institutions are to our health, economy, and security. United Nations, World Bank Group, and other global and regional institutions' concerted, cohesive response to the crisis's socioeconomic repercussions. As noted above, international organizations must urge for more “unconventional monetary policies” integrated with fiscal stimulus in developing nations, providing them with policy latitude to determine how to do so. They should also push developing country leaders to create a central crisis office (64). We need international funding to limit the epidemic and protect the most vulnerable. International collaboration may improve results. The post-virus global economy will have slowed growth, increased fragility, and more division. The IMF, OECD, G7, G20, World Bank, and Regional Development Banks must back focused, efficient, and proven-to-work measures in economies that need them to deal with the health, economic, employment, and social effects of the Pandemic on workers in all sectors of the economy, including self-employed and non-permanent, casual, and informal workers, and all businesses, tiny and medium-sized ones (SMEs). The world economy needs to change right away in the real world.

### 4.8. Getting and making investments easier

The term “foreign direct investment,” abbreviated as “FDI,” has emerged as essential in providing developing nations with financial resources, employment, advanced technology, and managerial expertise. Several underdeveloped countries have reaped significant dividends from these investments in accelerated economic growth and improved overall quality of life. Foreign direct investment could be a big part of the money needed to reach the Sustainable Development Goals by 2030 in areas like basic infrastructure, food security, preventing and adapting to climate change, and health and education (65). To achieve this goal, governments need to improve the efficiency with which they attract private investment, direct that investment toward sustainable development sectors, and make the most of the economic, social, and environmental benefits that result from that investment.

### 4.9. Sector-specific policy implications

Upon the onset of the crisis, many economists felt unable to respond. The globally synchronized halt in economic growth, particularly in manufacturing and commerce that preceded the viral breakout was made possible by currency flexibility and consistent government backing (62). In addition to liquidity and credit market difficulties, the need for supply-side information has encouraged financial analysts to acquire this understanding. If a person loses their job, they might not be able to pay their mortgage or rent before banks offer credit deferment or a way to get money. Mortgage fraud could hurt the market for mortgage-backed securities, mortgage funds, and the economy's growth (60). Losses on the financial markets happen worldwide and pull people's wealth, especially pensioners with fixed incomes and people who own securities. Investors in mortgage-backed securities have sold some of their shares. Markets haven't always been able to handle traditional policy tools like financial aid. With this instability, it's hard to know how to fix the world economy.

1. Trust and demand recovery necessitate supporting macroeconomic measures in this situation. Employers may use the ETC to pay salaries. Companies may benefit from interest-free loans to offset lost revenue.
2. Despite its flaws, the healthcare business has earned a lot of money mass-producing masks, hand sanitizers, and surgical equipment. Another recession-hit company might make medical equipment. It lacks the required hospitals and quarantine facilities. Updating and dispersing medical facilities may reduce viral transmission.
3. Online buying is trendy and will rise despite the uncertain economy. COVID-19 has natural and long-lasting repercussions for social contact and entertainment, making the tourist business obsolete and leaving many jobless.

## 5. Conclusion

Multiple industries may be impacted by a disease outbreak, including the capital market, labor market, foreign trade, consumer spending, and production. Because of the lockdown and the risk of spreading the disease, it's taking longer to make things people need. The products' supply chain has been broken, and national and international businesses will lose money. Revenue growth is slowing down because of poor cash flow in the market. There have been thousands of job losses due to the shutdown of industries. A disruption of the production process in several sectors has also negatively affected the GDP of many countries. This article demonstrates the global economic impact of the COVID-19 Pandemic in a straightforward but vivid manner. The virus has claimed thousands of lives and posed considerable challenges to countries. Bloomberg economists say it's too early to know the disease's full impact because it hasn't reached its peak yet. Capital economists estimate that if urgent action is not taken to reduce Wuhan 2019-nCoV as soon as possible, the first quarter of this year can bring China losses of up to $ 62 billion. In comparison, the world will suffer over $280 billion. The World Bank estimates that even a weak influenza pandemic, such as the H1N1 outbreak of 2009, could reduce global GDP by half, or about $300 billion.

### 5.1. Future study recommendation

It is the right time and place to study the effects of COVID-19 on the global economy and monetary policy of different countries. Even though there are some problems, the research on COVID-19 may lead to many good things. This could lead to new ideas and tests. In light of the COVID-19 crisis, the hidden assumptions in current research need to be looked at separately. The present study provides a roadmap on the literature tying the COVID-19 Pandemic to worldwide harm, directing future research on this new topic. COVID-19 receives regular media attention, although its relationship to monetary and public policy literature is weak. Researchers and policymakers must work together to create additional COVID-19 research. It would assist handle current and future problems. COVID-19 has complicated economic, health, and societal repercussions, making it a sensitive subject. This article focuses on the essential concerns and issues related to COVID-19 to help scholars and policymakers comprehend its worldwide influence on diverse industries.

## Author contributions

SN and KA contributed the ideas of the original draft. SN and SK wrote the introduction, literature review, and empirical outcomes sections. SP, HS, and MA helped to collect and visualize data of observed variables. SN, HS, and KA constructed the methodology section in the study. All authors contributed to the article and approved the submitted revised version.
